# Growth Kinetics of *Bacillus cereus* Vegetative Cells and Spores in Glutinous Rice Dough at Various Environmental Temperatures During Production

**DOI:** 10.1155/ijfo/1180461

**Published:** 2025-09-23

**Authors:** Zijie Dong, Xiao Zhang, Xiaojie Wang, Zhen Li, Huiping Fan, Biao Suo

**Affiliations:** ^1^ College of Food Science and Technology, Henan Agricultural University, Zhengzhou, China, henau.edu.cn; ^2^ Food Laboratory of Zhongyuan, Luohe, China; ^3^ Henan Qinre Industrial Holding Group Co., LTD, Xinxiang, China; ^4^ Key Laboratory of Staple Grain Processing, Ministry of Agriculture and Rural Affairs, Zhengzhou, China, agri.gov.cn; ^5^ National R&D Center for Frozen Rice & Wheat Products Processing Technology, Henan Engineering Research Center of Quick-Frozen Flour-Rice Food and Prepared Food, Henan Agricultural University, Zhengzhou, China, henau.edu.cn

**Keywords:** *Bacillus cereus*, glutinous rice foods, predictive modeling, spores, threshold of food safety risk

## Abstract

The growth and proliferation of *Bacillus cereus* in the processing environment are important reasons why the cell number in the final glutinous rice product exceeds the risk threshold. This study investigated the growth kinetics of *B. cereus* vegetable cells and their spores in glutinous rice dough at constant temperatures ranging from 11°C to 37°C. The results indicated that the Baranyi, modified Gompertz, and Huang models all successfully described the growth curves of the *B. cereus* vegetative cells and spores in glutinous rice dough, whereas the modified Gompertz model showed the best fitting accuracy in the majority of cases. The secondary Huang square root model successfully described the effects of temperature on the growth parameters of *B. cereus* vegetative cells and spores. This study revealed that, compared with spores, vegetative *B. cereus* cells had faster growth rates, shorter lag times, and higher concentrations (≥ 0.7 log CFU/g) than spores did. However, as the environmental temperature increased, the difference in the growth kinetics between the vegetative cells and spores gradually decreased, indicating that the residual spores in food at relatively high temperatures also have a considerable effect on food safety. Finally, an exponential model was regressed to fit the time required for *B. cereus* in glutinous rice dough to reach the critical threshold of food safety risk of 5 log CFU/g. The modeling of *B. cereus* growth in glutinous rice dough provides a theoretical basis for optimizing processing procedures to prevent exceeding the threshold before quick freezing of glutinous rice foods.

## 1. Introduction

Foodborne pathogens, including the spore‐producing *Bacillus cereus*, are the leading cause of human infection with foodborne diseases and food poisoning. Compared with vegetative cells, spores are more resistant to harsh environments, such as drying, heat, radiation, and chemical treatment, and the potential for foodborne diseases caused by spores is far greater than that caused by vegetative cells [1]. *B. cereus* is widely distributed in all kinds of foods, especially rice foods. The main reason is that *B. cereus* spores in the soil often contaminate harvested rice and survive and germinate during the cooking process [2, 3]. Moreover, many emetic toxins are produced in suitable environments, which are highly harmful to human health [4].

Rice is the most important food crop in East Asia and Southeast Asia, including China. Glutinous rice is a variety with strong viscosity because of its lack of amylose in the starch and its abundant amylopectin starch. Glutinous rice can be ground into dough and used to produce a variety of food, such as glutinous rice balls and rice cakes [5]. With the accelerated development of industrial production of glutinous rice food, ensuring its edible safety is particularly important. The food production workshop is an important place for biofilm adhesion, cross‐contamination, and multiplication of harmful microorganisms, and it is also one of the main causes of the excessive presence of harmful microorganisms in the produced foods [6–8]. Recent research reports indicate that in the process of grinding glutinous rice to make rice balls, *B. cereus* is ubiquitous in the factory environment, posing a serious hidden danger to food safety [9]. Improving the prevention and control of *B. cereus* in the processing and storage of glutinous rice dough is highly important to ensure food safety. This is because during the processing of glutinous rice foods, enterprises often cannot complete the entire processing process in a short period. The temporary preservation of semiproducts, mainly in the form of glutinous rice dough, is a common phenomenon. Owing to large‐scale food processing workshops, to save energy costs, enterprises often do not have good constant‐temperature control systems. Therefore, the temperature of the production environment is strongly affected by the season and the external environment, which greatly affects the growth and reproduction of microorganisms [10]. However, at present, the proliferation pattern of *B. cereus* vegetative cells and spores in glutinous rice dough is unclear and is unable to guide the establishment of glutinous rice food safety technologies.

During the industrial processing of glutinous rice foods, the final products are often quick‐frozen and then stored under freezing conditions. This procedure does not inactivate bacteria and is also an important reason for the repeated detection of *B. cereus* in quick‐frozen foods [11]. Therefore, the degree of proliferation of vegetative cells or spores of *B. cereus* contaminating raw materials has an important impact on the microbiological safety of the final products after quick freezing. The microbial growth kinetic model is widely used in predicting the shelf life of food, establishing the HACCP system, and assessing food safety risk [12]. By constructing an appropriate growth model to mathematically describe its growth pattern, the growth of the microorganisms contained in food can be predicted after the food experiences different environments; thus, the remaining shelf life of the food can be predicted [13]. A recent report indicated that the combination of the no‐lag phase model and the Huang square root model (HSRM) was suitable for describing the growth of mesophilic *B. cereus* in liquid egg yolk (LEY) [14]. According to the principle of a predictive microbial model, the growth curve of *B. cereus* in glutinous rice dough was expected to be used to assess the risk exceeding the standard during processing, which is beneficial for ensuring food safety in the production of quick‐frozen glutinous rice products. However, this type of predictive model has not been reported.

This study used a primary growth model and a secondary model to describe the growth kinetics of *B. cereus* vegetative cells and spores in glutinous rice dough at 11°C–37°C. A model was subsequently developed to describe the time required for *B. cereus* to reach 5 log CFU/g. The results can provide a reference for glutinous rice–producing factories to establish corresponding manufacturing procedures to prevent *B. cereus* from exceeding the risk threshold.

## 2. Methodology

### 2.1. Preparation of Bacterial Suspensions and Spores

The *B. cereus* ATCC 14579 and ATCC 7039 strains were sourced from the American Type Culture Collection and stored in glycerol tubes at −80°C. One loop of the culture from a single colony of each strain was inoculated into 10 mL of tryptic soy broth with 0.6% yeast extract (TSB‐YE) (Land Bridge Technology Co. Ltd., Beijing, China). The cells of the two *B. cereus* strains were incubated separately at 37°C for 8 h until the late exponential stage (approximately 10^9^ CFU/mL). The cells were washed three times with sterile peptone water and collected as stock cultures of vegetative cells. One hundred microliters of the late exponential cells were spread on tryptone soy agar (TSA + YE) containing 0.05 g/L MnSO_4_, which was used to induce spore formation [15]. After aerobic culture at 37°C for 7 days, the surface of the agar was flooded with 3 mL of 0.1% sterile peptone water (Land Bridge Technology Co. Ltd., Beijing, China). The bacterial lawn was scraped off with a sterile coating stick and then transferred to a sterile centrifuge tube. The tubes were placed in a water bath at 80°C for 10 min to kill the vegetative cells. After washing three times with sterile peptone water, the spores were obtained by centrifuging at 4000 × g for 10 min at 4°C [16]; the spores were subsequently suspended in sterile peptone water and stored at 4°C for use as stock spore cultures.

### 2.2. Preparation of Glutinous Rice Dough and Bacterial Inoculation

Prior to dough preparation, glutinous rice flour (Yihai Kerry Food Industry Co. Ltd., Zhengzhou, China) was subjected to UV sterilization for 30 min to inactivate the background flora. The glutinous rice flour and sterile water were mixed at a ratio of 5:4 (g/mL) and kneaded evenly to prepare the dough. Based on the processing experience of the factory, the glutinous rice dough made with this flour–water ratio is suitable for being processed into traditional Chinese representative glutinous products such as tangyuan. The pH of the prepared dough was 6.1, and the water activity was 0.94. Five grams of the dough were packaged in a sterile homogeneous bag (12 × 18 cm, Qingdao Hi‐Tech Industrial Park Haibo Biotechnology Co. Ltd.). The suspensions of late exponential cells from the two *B. cereus* strains were mixed at equal concentrations to prepare the working inoculants of vegetative cells. The spore granules were also mixed to prepare a working spore suspension. The two suspensions were serially diluted with 0.85% sterile normal saline. One hundred microliters of the diluted bacterial mixture were inoculated evenly on the surface of the glutinous rice dough to a final concentration of approximately 3 log CFU/mL. The entire preparation was performed under a sterile workbench.

### 2.3. Enumeration of Bacterial Cells

First, the inoculated glutinous rice dough was placed in the sterile homogeneous bag, then stored at constant temperatures of 4°C, 11°C, 18°C, 25°C, 32°C, and 37°C in incubators (LRH‐150F, Yiheng Technology Co. Ltd., Shanghai, China). According to enterprise investigations, 11°C–37°C is the common environmental temperature range of glutinous rice dough production in the winter and summer production chains. The samples were collected at predefined time intervals to enumerate the viable bacterial cells. Each sample was added to 45 mL of 0.85% sterile normal saline, and the samples were homogenized with a Stomacher Lab blender (Model 3500, Seward Laboratories, London, United Kingdom) by beating 12 times/s for 2 min. After 10‐fold serial dilution, 100 *μ*L of the sample mixture was spread onto mannitol–yolk polymyxin (MYP) agar basal plates (Land Bridge Technology Co. Ltd., Beijing, China) and incubated at 37°C for 24 h for viable *B. cereus* cell enumeration.

### 2.4. Construction of the Primary Growth Model of *B. cereus* in Glutinous Rice Dough

The Huang [17], Baranyi [18], and modified Gompertz [19] models were used to regress the growth curve of *B. cereus* in glutinous rice dough at various temperatures. The Huang models are shown in Equations (1) and (2), the Baranyi models are shown in Equations (3) and (4), and the modified Gompertz model is shown in Equation (5).

(1) Yt=Y0+Ymax−lneY0+eYmax−eY0e−μmaxBt,

(2) Bt=t+1αln1+e−αt−λ1+eαλ,

(3) Yt=Y0+μmaxAt−ln1+expμmaxAt−1expYmax−Y0,

(4) At=t+1μmaxlnexp−μmaxt+exp−h0−exp−μmaxt−h0,

(5) Yt=Y0+Ymax−Y0exp−expμmaxeYmax−Y0λ−t+1,

where *Y*(*t*) is the number of colonies at time *t* (log CFU/g), *Y*_0_ and *Y*_max_ are the initial number of bacteria and the number of bacteria in the stable phase (log CFU/g), *μ*_max_ is the maximum specific growth rate (h^−1^), *t* is the culture time (hours), *λ* is the lag time (hours), and *h*_0_ reflects the physiological state of bacteria, *h*_0_ = *λ* *μ*_max_. *α* is a constant (*α* = 4).

### 2.5. Construction of the Secondary Growth Model

HSRM [20] and Ratkowsky square root model [21] were used as secondary models to evaluate the effect of temperature on the growth rate of *B. cereus* in glutinous rice dough. The Huang and Ratkowsky models are shown in Equations (6) and (7), respectively.

(6) μmax=aT−Tmin0.751−expbT−Tmax,

(7) μmax=aT−T01−expbT−Tmax,

where *T*_min_ and *T*_max_ represent the minimum and maximum growth temperatures (degrees Celsius), respectively; *T*_0_ represents the minimum growth temperature (degrees Celsius); *T* represents the culture temperature (degrees Celsius); and *μ*_max_ represents the maximum specific growth rate (h^−1^).

An exponential model in Equation (8) was used to estimate the time needed to reach 5 log CFU/g (*t*_5.0_) in glutinous rice dough.

(8) t5.0=aebT.

In this equation, *T* represents the environmental temperature. The values of *t*_5.0_ for model regressing were calculated according to the most appropriate primary model at each treatment temperature (*T*).

### 2.6. Model Validation

For validation of the primary and secondary models, the kinetic parameters obtained from the models were applied to predict bacterial growth at different environmental temperatures. Moreover, external validation was designed on the basis of the growth data obtained at a constant temperature of 20°C. The correlations between the measured values and the predicted values were compared to confirm the fit of the model. One‐step kinetic analysis was used in this study to estimate the kinetic parameters via the USDA Integrated Pathogen Modeling Program (IPMP) – Global Fit [22]. To compare the effectiveness of different models, the Akaike information criterion (AIC) of each model was calculated with respect to the bacterial counts [23]. The coefficient of determination (*R* ^2^) and root mean square error (RMSE) were used to assess the adequacy of the internal and external validation of the developed predictive growth models. Moreover, the accuracy factor (*A*_*f*_) and bias factor (*B*_*f*_) were applicable for secondary model evaluation. *A*_*f*_ indicates the absolute value of the mean difference between the observed values obtained through the experiment and the predicted values of the secondary model. In contrast, *B*_*f*_ indicates the structural bias of the model [24]. The values of *R* ^2^, *A*_*f*_, *B*_*f*_, and RMSE were calculated according to Equations (9)–(12).

(9) R2=1−∑Pdi−Obi2∑Obi−The average of the Obi2,

(10) RMSE=∑Obi−Pdi2n,

(11) Af=10∑IgObi/Pdin,

(12) Bf=10∑IgObi/Pdin,

where *P* *d* *i* is the value predicted by the model and *O* *b* *i* is the experimental observations.

## 3. Results and Discussion

### 3.1. Construction of the Primary Growth Model of *B. cereus* in Glutinous Rice Dough

**Figure 1:**
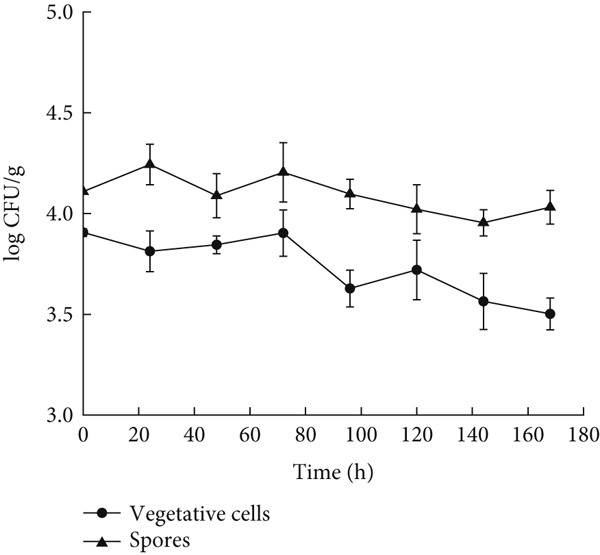
Growth curves of vegetative cells and spores in glutinous rice dough at 4°C.

When glutinous rice dough was inoculated with vegetative *B. cereus cells*, the number of viable cells slowly decreased at 4°C, with a decrease of 0.4 log CFU/g (*p* < 0.05) within 180 h, and each set of experiments was repeated at least three times to ensure the accuracy of the results. However, when spores were inoculated into glutinous rice dough, the number of viable cells that could be cultured did not decrease significantly. Compared with *B. cereus* vegetative cells, spores are more resistant to low temperatures. However, these two types of cells do not fit into the primary growth model (Figure 1). This result is similar to that of liquid milk in that *B. cereus* can only grow at ambient temperatures higher than 4°C [25]. When the ambient temperature was between 11°C and 37°C, *B. cereus* in glutinous rice dough grew, and the Huang, Baranyi, and modified Gompertz models were used to describe the growth kinetics.

**Figure 2:**
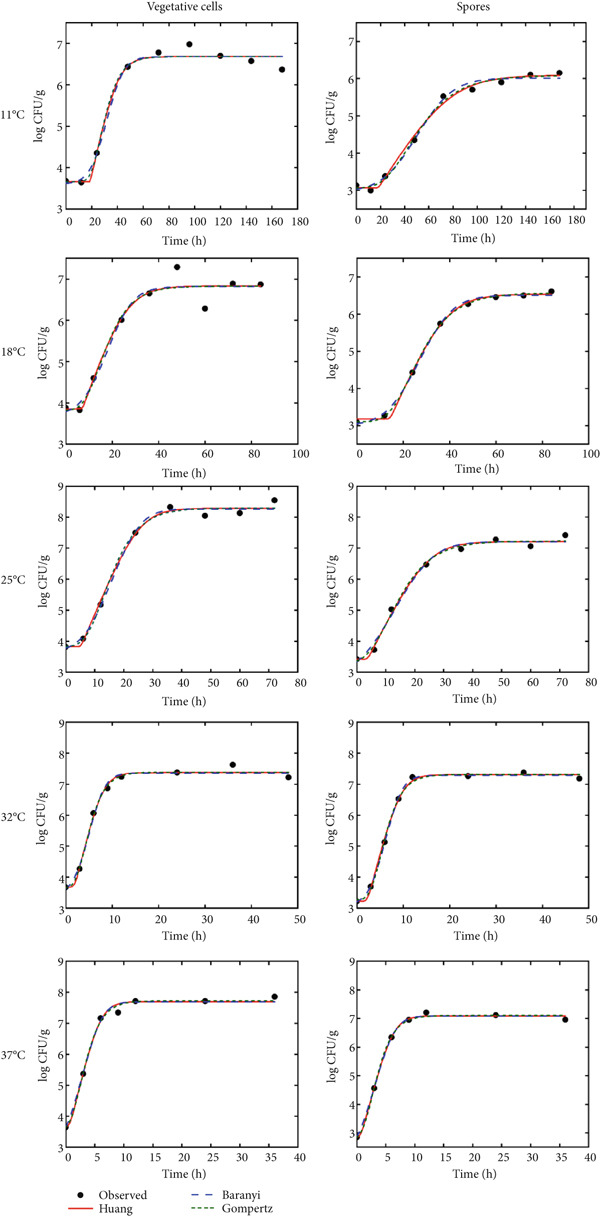
Primary growth model of *B. cereus* vegetative cells and spores in glutinous rice dough at different temperatures.

As shown in Figure 2, the experimental values of *B. cereus* in the glutinous rice dough at each temperature ranging from 11°C to 37°C were close to the values predicted by the corresponding models, indicating that the three models all successfully described bacterial growth. With increasing temperature, the lag phase of the vegetative cells and spores in glutinous rice dough gradually decreased, whereas the growth rate gradually increased. When the inoculated vegetative cells reached the stable growth phase, the maximum viable cell number was 7.79 log CFU/g, which was significantly greater than the 7.13 log CFU/g determined for inoculated spores (*p* < 0.05).

### 3.2. Comparative Analysis of the Primary Models

The indicators of lag time *λ* (hours) and maximum specific growth rate *μ*_max_ (h^−1^) were used to determine the microbial growth rate. AIC, MSE, RMSE, and *R* ^2^ were used to evaluate the models. The closer *R* ^2^ is to 1, the better the fitting result of the model at this temperature. A model with lower AIC, MSE, and RMSE values is better than one with higher values [26].

When the glutinous rice dough was inoculated with *B. cereus* vegetative cells, the values of AIC, MSE, RMSE, and *R* ^2^ obtained by fitting the Huang, Baranyi, and modified Gompertz models were similar (Table 1), indicating that these three models are effective for glutinous rice with good fitting accuracy. When glutinous rice dough was inoculated with spores, the fitting accuracy parameters of the three primary growth models greatly differed. The AIC values of the modified Gompertz model at 4°C were lower than those of the other models. A comparison of the other fitting accuracy parameters also revealed that the germination and growth curves of spores in glutinous rice dough at temperatures ranging from 11°C to 37°C were more suitable for fitting with the modified Gompertz model. All the models performed well at mild temperatures (18°C–32°C), which may constitute the ideal growth temperature range for *B. cereus* [1], allowing its growth to be more predictable. Previous studies revealed that the Huang, Baranyi, and modified Gompertz models all described well the growth curves of *S. aureus* (10°C–43°C) [24] and *B. cereus* (13°C–46°C) [2] in rice products, with no significant difference in fitting degree.

**Table 1:** Growth kinetic parameters of *B. cereus* vegetative cells and spores in glutinous rice dough at different temperatures.

**Temperature**	**Cell physiological state**	**Model**	**λ** **(h)**	**μ**_max_ **(h** ^**−1**^ **)**	**AIC**	**MSE**	**RMSE**	**R** ^2^
11°C	Vegetative cells	Huang	18.976	0.148	−3.841	0.030	0.174	0.9961
		Baranyi	23.141	0.178	−3.423	0.032	0.178	0.9951
		Modified Gompertz	19.007	0.136	−3.767	0.030	0.174	0.9959
	Spores	Huang	22.251	0.057	−10.144	0.015	0.121	0.9913
		Baranyi	36.795	0.093	−9.036	0.017	0.129	0.9946
		Modified Gompertz	27.298	0.059	−13.066	0.011	0.104	0.9951
18°C	Vegetative cells	Huang	7.161	0.163	4.615	0.078	0.278	0.9542
		Baranyi	10.886	0.210	5.266	0.083	0.288	0.9510
		Modified Gompertz	7.372	0.151	5.080	0.080	0.283	0.9524
	Spores	Huang	14.384	0.163	−5.284	0.004	0.062	0.9986
		Baranyi	16.971	0.170	−4.408	0.004	0.065	0.9988
		Modified Gompertz	13.492	0.128	−16.438	0.001	0.030	0.9998
25°C	Vegetative cells	Huang	5.227	0.223	9.832	0.026	0.162	0.9960
		Baranyi	7.890	0.259	9.056	0.024	0.155	0.9971
		Modified Gompertz	6.635	0.252	9.124	0.024	0.155	0.9964
	Spores	Huang	3.771	0.187	6.821	0.018	0.132	0.9955
		Baranyi	4.764	0.199	10.224	0.027	0.165	0.9915
		Modified Gompertz	3.357	0.184	6.650	0.017	0.130	0.9952
32°C	Vegetative cells	Huang	1.935	0.624	6.186	0.017	0.129	0.9944
		Baranyi	2.634	0.733	8.588	0.023	0.152	0.9909
		Modified Gompertz	1.997	0.605	4.754	0.014	0.120	0.9957
	Spores	Huang	2.285	0.574	−0.118	0.008	0.088	0.9982
		Baranyi	3.380	0.711	−5.950	0.004	0.062	0.9994
		Modified Gompertz	2.798	0.608	1.863	0.010	0.099	0.9977
37°C	Vegetative cells	Huang	0.651	0.786	40.788	0.015	0.124	0.9929
		Baranyi	0.825	0.809	40.955	0.016	0.128	0.9925
		Modified Gompertz	0.651	0.786	38.747	0.012	0.109	0.9945
	Spores	Huang	1.887	0.615	37.865	0.010	0.099	0.9959
		Baranyi	2.473	0.695	41.425	0.017	0.129	0.9931
		Modified Gompertz	2.020	0.623	36.680	0.009	0.093	0.9964

As shown in Table 1, at 11°C–37°C, according to the calculation results of the modified Gompertz model, the maximum specific growth rate (*μ*_max_) of *B. cereus* in glutinous rice dough inoculated with vegetative cells and spores increased with increasing temperature from 0.136 and 0.059 h^−1^ to 0.786 and 0.623 h^−1^, respectively, whereas the corresponding lag time (*λ*) decreased from 19.007 and 27.298 h to 0.651 and 2.020 h, respectively. At lower temperatures (11°C–25°C), the *μ*_max_ of the vegetative cells of *B. cereus* was significantly greater than that of the spores, and the *λ* was significantly lower at the corresponding temperature. However, at relatively high temperatures (32°C–37°C), the difference in *μ*_max_ between the vegetative cells and the spores was not significant, but the *λ* of the vegetative cells was still lower than that of the spores at the same temperature. These findings indicate that, compared with vegetative *B. cereus cells*, spores have greater low‐temperature resistance but germinate in glutinous rice dough for a longer period of time and grow more slowly after germination [27, 28]. With increasing ambient temperature, spores germinate and grow at a rate gradually close to that of vegetative cells.

### 3.3. Construction and Comparison of Secondary Models of *B. cereus* in Glutinous Rice Dough

The modified Gompertz model was chosen as the most appropriate primary growth model for describing the growth of *B. cereus* vegetative cells and spores in glutinous rice dough. Two secondary models, the Ratkowsky square root model and the HSRM, were used to describe the relationship between the storage temperature and growth rate (Figure 3). Both secondary models successfully described the effect of temperature on the growth rate of *B. cereus* in glutinous rice dough inoculated with vegetative cells and spores (Table 2). The growth rate and lag phase of *B. cereus* exhibited a logarithmic linear relationship when the modified Gompertz model was used as a primary model (Figure 4). Similarly, the Ratkowsky equations were also successfully employed as the secondary model for describing the *μ*_max_ and *λ* values associated with the growth of Shiga toxin‐producing *Escherichia coli* in Minas Frescal cheese [29]. The fitting degree of *B. cereus* spores was lower than that of the other two cases, indicating that the linear relationship between the growth rate and the lag phase of spores under these model fitting conditions was lower than that of vegetative cells. This may be related to the poorer low‐temperature germination of spores [30].

Figure 3Fitting curves of the two secondary models, Ratkowsky and Huang, which describe the growth rates of *B. cereus* (a) vegetative cells and (b) spores in glutinous rice dough samples at different temperatures.

**Figure:**
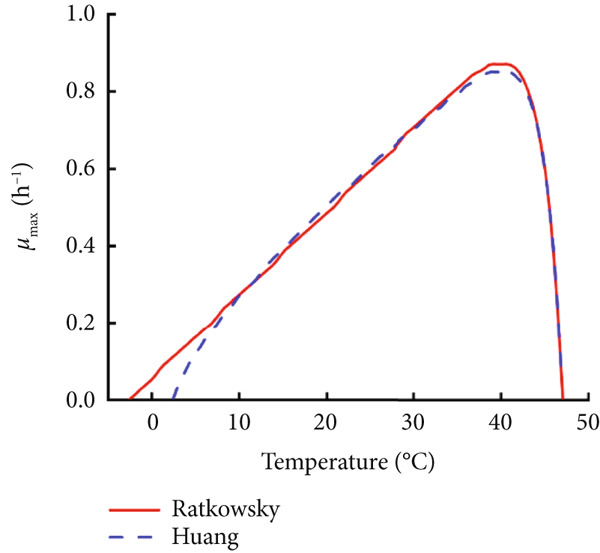
(a)

**Figure:**
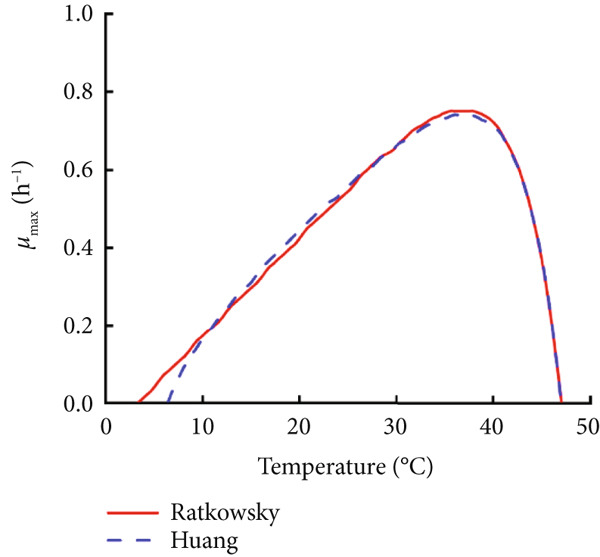
(b)

**Table 2:** The fitting parameters of the secondary model of *B. cereus* in glutinous rice dough.

**Cell physiological state**	**Model**	**AIC**	**A**_**f**_	**B**_**f**_	**MSE**	**RMSE**	**R** ^2^
Vegetative cells	Ratkowsky	29.191	1.098	0.993	0.003	0.057	0.9510
	Huang	30.946	1.110	1.000	0.004	0.064	0.9391
Spores	Ratkowsky	−5.998	1.092	0.990	0.004	0.060	0.9279
	Huang	−4.330	1.116	0.989	0.005	0.068	0.9080

Figure 4Linear relationship between *λ* and *μ*_max_ in the primary model of *B. cereus* (a) vegetative cells and (b) spores in glutinous rice dough at different temperatures.

**Figure:**
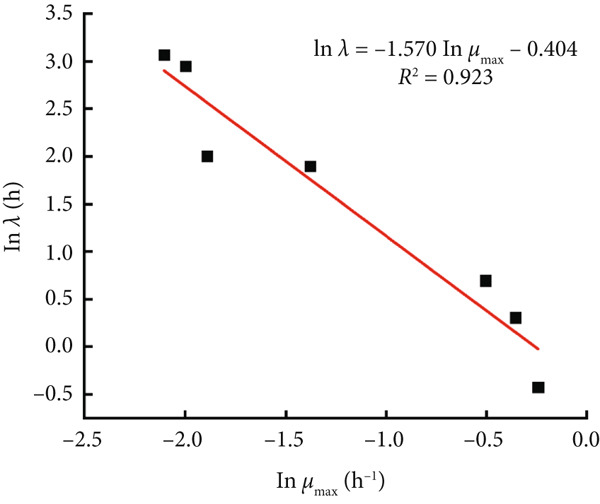
(a)

**Figure:**
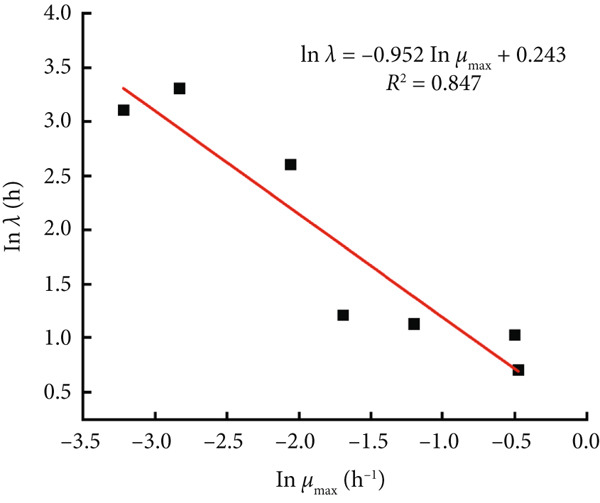
(b)

The minimum and maximum growth temperatures of the vegetative cells and spores in the glutinous rice dough fitted by the Ratkowsky model were −2.6°C and 3.4°C and 47.1°C and 47.1°C, respectively, whereas those of the Huang model were 2.4°C and 6.5°C and 47.1°C and 47.0°C, respectively (Table 3). Studies have shown that the minimum, optimum, and maximum growth temperatures of *B. cereus* in cooked rice are 8.2°C, 37.6°C, and 46.8°C [31], respectively. In a previous study, the lowest temperature for psychrotrophic *B. cereus* at which “growth” was observed is 5°C [32]. In this study, neither vegetative cells nor spores grew at 4°C, and the number of vegetative cells even decreased to some extent (Figure 1). Therefore, the deviation of the lowest growth temperature between the values predicted via the Ratkowsky model (−2.6°C and 3.4°C) and the deviation of the measured values (≥ 4°C) was more significant. Therefore, the HSRM was more suitable as the secondary model of *B. cereus* vegetative cells and spores in glutinous rice dough, although the prediction of the lowest growth temperature was still unsatisfactory.

**Table 3:** Coefficients of secondary models for the specific growth rate of *B. cereus* in glutinous rice dough.

**Models**	**Parameters**	**Estimated values**	**Standard error**	**t** **value**	**p** **value**
Vegetative cells					
Ratkowsky	*a*	0.022	0.004	5.123	1.440 × 10^−2^
	*b*	0.424	0.131	3.238	4.793 × 10^−2^
	*T*_min_	−2.568	5.452	−0.471	0.670
	*T*_max_	47.108	0.203	231.713	1.773 × 10^−7^
Huang	*a*	0.059	0.010	6.084	8.916 × 10^−3^
	*b*	0.463	0.162	2.860	6.459 × 10^−2^
	*T*_0_	2.429	4.692	0.518	0.641
	*T*_max_	47.105	0.225	209.718	2.391 × 10^−7^
Spores					
Ratkowsky	*a*	0.026	0.006	4.386	1.182 × 10^−2^
	*b*	0.206	0.075	2.759	5.092 × 10^−2^
	*T*_min_	3.397	4.128	0.823	0.457
	*T*_max_	47.056	0.316	149.043	1.216 × 10^−8^
Huang	*a*	0.063	0.011	5.741	4.562 × 10^−3^
	*b*	0.238	0.089	2.672	5.569 × 10^−2^
	*T*_0_	6.475	3.283	1.972	0.120
	*T*_max_	47.046	0.339	138.692	1.621 × 10^−8^

### 3.4. External Validation of the Models

To validate the regression models, the modified Gompertz model was used to describe the growth of *B. cereus* vegetative cells and spores in glutinous rice dough at a constant temperature of 20°C, and the experimental values were compared with the regression models. The predicted values were visually compared (Figure 5). Model validation revealed that for each of the abovementioned vegetative cells and spores, RMSE was less than 0.29, and *R* ^2^ was greater than 0.99, indicating that the modified Gompertz model could accurately predict the growth of *B. cereus* in glutinous rice dough.

Figure 5Correlations between the observed values and predicted values at 20°C for the external experiments. The glutinous rice dough was inoculated with (a) vegetable cells or (b) spores.

**Figure:**
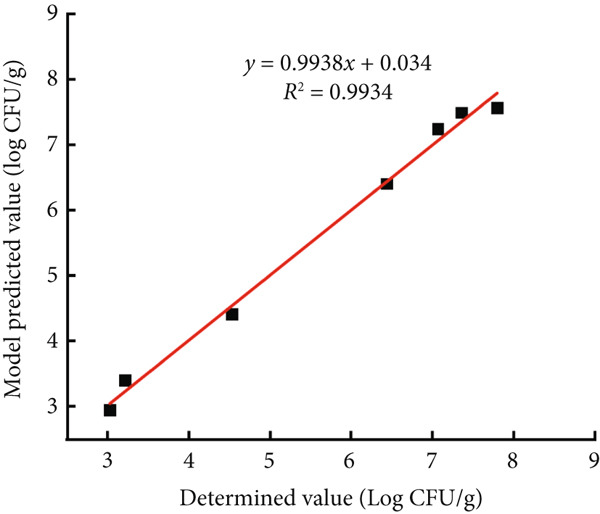
(a)

**Figure:**
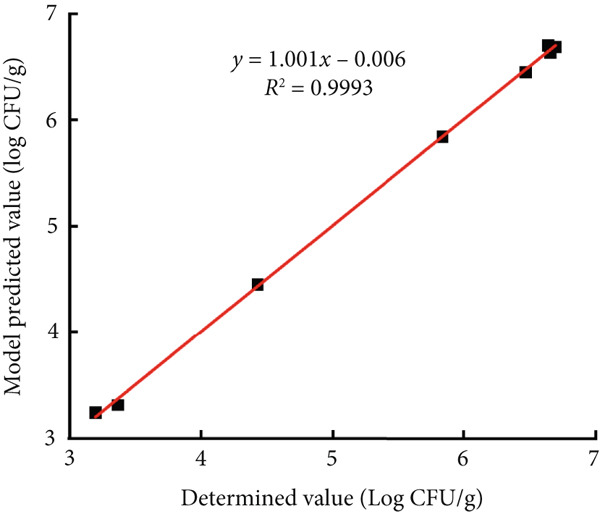
(b)

Epidemiological evidence suggests that the majority of outbreaks worldwide caused by *B. cereus* are associated with concentrations higher than 10^5^ CFU/g in implied foods [33]. Moreover, the time to reach 5 log CFU/mL *B. cereus* in the dairy mixture was correlated with the time to first cereulide quantification [34]. Therefore, on the basis of the primary modified Gompertz fit in Table 1, the time required for the proliferation of *B. cereus* in glutinous rice dough to reach the level of 5 log CFU/g at 11°C–37°C was calculated, and an exponential model was regressed in Equations (13) and (14):

(13) Vegetative cells:t5.0=86.373 e−0.093T.

(14) Spores:t5.0=217.18 e−0.113T.

**Figure 6:**
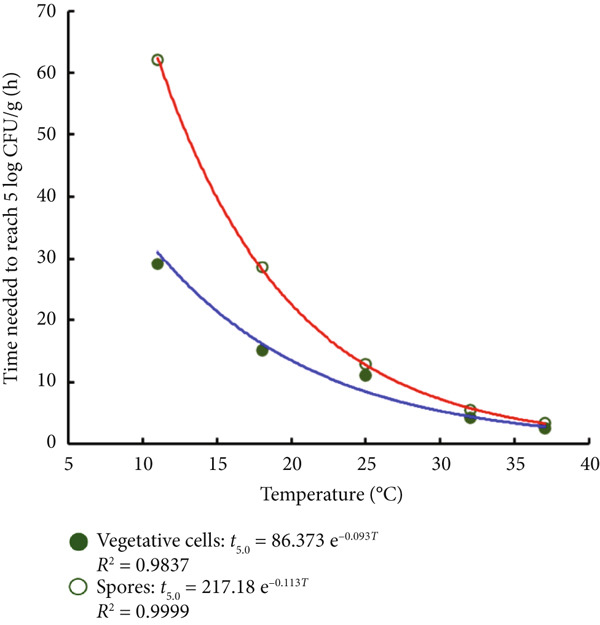
Exponential models used to describe the time needed to reach 5.0 log CFU/g of *B. cereus* vegetative cells and spores in glutinous rice dough.

The exponential model effectively simulated the growth time for *B. cereus* vegetative cells and spores to reach 5.0 log CFU/g at specific ambient temperatures ranging from 11°C to 37°C. Figure 6 shows that at 11°C, the time required for the growth of vegetative bodies and spores to reach 5 log CFU/g was the greatest, with values of 29.1 and 62.1 h, respectively. The difference in the required time gradually decreased with increasing ambient temperature, which was 2.5 and 3.4 h at 37°C. The results of this study were similar to those reported for *Bacillus subtilis*; a lower ambient temperature was unfavorable for spore germination [35]. The results suggest that glutinous rice food processing enterprises should attempt to maintain the production environment at a relatively low temperature during the production process. When ambient temperature control conditions do not allow this, glutinous rice food processing enterprises should, on the basis of the status of *B. cereus* in glutinous rice dough, examine the status of vegetative cells or spores and develop scientific processing procedures [36] to ensure that the level does not exceed 5 log CFU/g before production is complete.

## 4. Conclusion

In this study, a growth kinetics model of the vegetative bodies and spores of *B. cereus* in glutinous rice dough was fitted under constant‐temperature conditions ranging from 11°C to 37°C. The results showed that the Baranyi model, modified Gompertz model, and Huang model fit the growth curves of the abovementioned vegetative cells and spores in glutinous rice dough well. However, in most cases, the modified Gompertz model showed the best fitting accuracy. The secondary HSRM successfully described the effects of temperature on the growth parameters of *B. cereus* vegetative cells and spores. This study subsequently used an exponential model to fit the exponential model of the time required for *B. cereus* in glutinous rice dough to reach the critical food safety risk threshold of 5 log CFU/g. The model provides a basis for designing production processes for glutinous rice food processing enterprises at different environmental temperatures.

## Conflicts of Interest

The authors declare no conflicts of interest.

## Author Contributions

Zijie Dong: methodology, investigation, and writing—original draft. Xiao Zhang: investigation and software. Xiaojie Wang: data curation. Zhen Li: validation and funding acquisition. Huiping Fan: software and visualization. Biao Suo: conceptualization, funding acquisition, supervision, project administration, and writing—review and editing.

## Funding

This study was funded by the National Natural Science Foundation of China (32272441), the Key Project of Henan Natural Science Foundation (242300421201), and the Natural Science Foundation of Henan Province (232300420008).

## Data Availability

The data that support the findings of this study are available from the corresponding author upon reasonable request.
